# How does nitrogen application impact soil respiration and CH_4_ emissions? An experiment in an oil palm plantation on tropical peat

**DOI:** 10.1098/rsos.252443

**Published:** 2026-09-09

**Authors:** Kristell Hergoualc'h, Louis Verchot, Satria Oktarita, Nelda Dezzeo

**Affiliations:** ^1^ Center for International Forestry Research (CIFOR), Jl. CIFOR, Situ Gede, Bogor 16115, Indonesia; ^2^ Centre de coopération International en Recherche Agronomique pour le Développement (CIRAD), UMR Eco&Sols, Montpellier, France; ^3^ Mazingira Centre for Environmental Research and Education, International Livestock Research Institute (ILRI), Nairobi, Kenya; ^4^ Center for International Tropical Agriculture (CIAT), Km 17, Recta Cali-Palmira, Cali, Colombia; ^5^ Yayasan Hima Alam Raya, Kabupaten Lima Puluh Kota, West Sumatra, Indonesia; ^6^ Venezuelan Institute of Scientific Research (IVIC), Caracas, Venezuela

**Keywords:** agriculture, climate change, CO_2_, global warming, greenhouse gas, peat decomposition, tropical peatland

## Abstract

Oil palm cultivation on peatlands causes severe environmental damage, yet its greenhouse gas (GHG) footprint and underlying biogeochemical drivers remain poorly quantified. This study assessed how nitrogen fertilizer application impacts soil respiration and CH_4_ emissions across spatio-temporal scales in a fertilization trial with three application rates: 0 (N0), 154.5 (N1) and 309 (N2) kg N ha^−1^ yr^−1^. Fluxes and environmental variables were monitored monthly over a year and daily for a month after two fertilizations. Measurements were taken close to palms (CP), where fertilizer was applied, and farther from palm (FP). Soil respiration (Mg CO_2_-C ha^−1^ yr^−1^) was high compared to the literature, with N0 and N2 (27.3 ± 2.8 and 28.5 ± 3.0) exceeding N1 (18.8 ± 2.5). Fertilization triggered short-lived respiration increases, without affecting annual rates. Treatment and spatial differences (FP > CP) aligned with soil temperature and soil N_2_O emission patterns, suggesting links to peat mineralization. CH_4_ emissions (kg CH_4_-C ha^−1^ yr^−1^) were greater in N0 (3.9±1.5) compared to N1 (0.7 ± 0.6) and N2 (1.0 ± 1.3), inversely mirroring soil nitrate distribution under unfertilized conditions. A transient fertilizer effect occurred, without affecting annual rates. Our findings highlight strong interactions between soil respiration, CH_4_ emissions and N cycling in drained tropical peatlands, warranting further investigation to constrain GHG process-based modelling.

## 1. Introduction

Oil crops are produced on over 300 million ha worldwide, and demand for edible oils is growing at around 4% per year, according to private sector market assessments [1]. The expansion of oil palm production, particularly in Southeast Asia, has contributed to the economic development of rural communities, but it has also generated significant controversy owing to its environmental impacts, particularly when it is produced on peatlands [2,3]. Malaysia and Indonesia began large-scale production in the 1960s, and Indonesia has been the largest producer of crude palm oil since 2005 [4]. In Indonesia, industrial plantations expanded by 6.2 Mha between 2001 and 2019, resulting in 2.1 Mha of deforestation [5]. Over the same period, smallholder plantations expanded by 2.3 Mha, resulting in an additional 0.7 Mha of deforestation. Plantation expansion slowed after 2012, declining more sharply in 2017, 2018 and 2019. In addition to deforestation, this expansion has also contributed to wildfire, haze and associated high levels of particulate matter (2.5), biodiversity loss and water supply disruption [6,7].

One of the more contentious issues with oil palm expansion is the conversion of organic soils, resulting in the loss of primary and secondary swamp forests. Draining ombrotrophic peatlands for palm production leads to progressive soil surface subsidence and large emissions of greenhouse gases (GHGs) [8,9]. This subsidence eventually causes flooding and salt water intrusion, rendering the land unsuitable for future production and making palm oil production unsustainable over the long term. The GHG footprint of this conversion is approximately 2200 Mg CO_2_ eq ha^−1^ over a typical 25-year plantation lifespan; about half of this is due to peat decomposition, approximately 26% to forest biomass loss and the remainder to fire [10].

There has been some development of our understanding of the impacts of deforestation for oil palm plantations on peat on soil respiration and methane (CH_4_) emissions. Soil respiration—the largest component of ecosystem respiration—stems from root respiration (autotrophic) and microbial respiration (heterotrophic) [11]. A meta-analysis found no consistent effect of peat swamp forest conversion on soil respiration [12], largely because autotrophic and heterotrophic respiration respond in opposite ways—autotrophic respiration decreases post-conversion, while heterotrophic respiration increases. Soil CH_4_ emissions result from antagonistic yet interconnected microbial processes. Methane is produced by methanogens in anaerobic sites during organic matter decomposition and consumed in aerobic sites via oxidation by methanotrophs [13]. Pathways for CH_4_ to transfer from wetland soil to the atmosphere include diffusion, ebullition and transport via the aerenchyma of plants. Tropical peat swamp forests are typically large CH_4_ sources, and their drainage and conversion to any land use, including oil palm plantations, reduce CH_4_ emissions [14]. However, this reduction never offsets the simultaneous increase in soil CO_2_ emissions owing to accelerated peat decomposition.

The main biogeochemical factors influencing soil respiration and CH_4_ fluxes in drained peatlands are temperature, soil water content (usually estimated via water table (WT) depth) and substrate quality [15–18]. Diel soil temperature variations are small in tropical peat swamp forests but increase following deforestation [19]. Intermittent shading in palm plantations can reduce soil temperature peaks locally, affecting the soil CO_2_ efflux from a plantation [20]. The role of soil water content as a regulator of GHG emissions in tropical peatland systems remains incompletely understood [15]. Shallow WTs generally reduce total soil respiration but stimulate CH_4_ emissions [16,18,21–23]. However, WT depth appears to have a limited influence on temporal soil respiration variability [24,25]. Few studies in drained tropical peatlands use the soil water-filled pore space (WFPS) as an explanatory variable for GHG dynamics [23,26]. This variable integrates both soil water content and the extent of the peat profile exposed to aerobic conditions. In upland systems, the WFPS has been a key parameter for explaining GHG fluxes since production and consumption processes typically occur in surface layers, and these layers also limit gas diffusion across the soil–atmosphere interface [27–29]. Substrate quality significantly affects organic matter decomposition, with the high C:N ratio of ombrotrophic tropical peat soils suggesting N limitation [17]. Conversion of peat swamp forest to plantation alters soil C:N ratios, either through declines in C concentration [19] or N concentration [30]. Such conversion degrades organic matter quality, with converted sites showing higher concentrations of aromatic and aliphatic compounds and lower polysaccharide content than intact forests [31]. Evidence suggests negative correlations between CH_4_ emissions and soil C:N ratios [32] and highlights recent plant residues as a major substrate for methanogens [13]. Fertilization further modifies these dynamics. Nitrogen additions can temporarily enhance soil respiration by stimulating root or microbial activity [33], though long-term studies in oil palm plantations on peat report no significant fertilization effect [34]. For CH_4_, nitrate (NO_3_^−^) inputs can decrease production by raising redox potentials [13], while ammonium (NH_4_^+^) competes with CH_4_ for the CH_4_ monooxygenase, thereby inhibiting CH_4_ oxidation [35].

Research on GHG fluxes in oil palm plantations on peat has largely focused on the effects of standard industry management practices [18,36], with relatively few studies experimentally testing alternative options or manipulating key drivers of GHG fluxes [16,34,37]. In addition, fertilizer in these systems is typically applied around palm bases rather than evenly across the soil surface. This spatial heterogeneity is often overlooked in surface flux studies, which can introduce bias into annual flux estimates [38]. In this context, this study aims to enhance understanding of how fertilization influences soil respiration and CH_4_ fluxes, considering responses across multiple spatio-temporal scales. We hypothesize that greater N availability from fertilization will stimulate both fluxes near palms, with little effect on overall annual soil CO_2_ and CH_4_ plantation efflux. We also expect both fluxes to be higher close to palms (CPs) based on the assumption of greater root respiration and C inputs at this spatial position. Finally, we test the hypothesis that the WFPS is a better predictor of the temporal variability in soil respiration than WT depth [34], whereas the reverse holds for CH_4_ fluxes, and soil temperature is not expected to be a significant control of temporal variation.

## 2. Methods

### 2.1. Site description

The study was conducted in an oil palm plantation in Arang Arang, Jambi District, Sumatra, Indonesia (1°38′S, 103°54′E). Long-term records from the airport climate station in Jambi indicate an average annual air temperature of 26.5°C and an average annual rainfall of 2466 mm, with June–August typically being the driest period [39]. The plantation sits on deep peat (in excess of 8.5 m) classified as Folic Hemic Histosol dystric drainic per IUSS Working Group WRB (2006) standards [40]. The site was cleared of native vegetation by fire in 2004 and planted with oil palms in December 2009 at a density of 149 palms ha^−1^ (spaced 8.8 m in a triangular arrangement). Additional land-clearing fires may have occurred between the initial clearance and planting, though no records exist. The peat surface is relatively flat owing to compaction during land preparation. A drainage system aims to maintain the WT at around 60 cm below soil surface to facilitate oil palm growth, although this target is not always achieved. The system comprises primary canals exceeding 400 cm depth, secondary canals reaching approximately 150 cm and tertiary canals approximately 75 cm deep.

### 2.2. Experimental plots

The experimental plots were part of an 11.7 ha long-term fertilization trial (depicted in supplementary material, §S1, supplementary material fig. S1), structured as a 3^3^ × 2 factorial design. The trial incorporated three levels of nitrogen (N0, N1 and N2 received as urea), phosphorus (P0, P1 and P2 received as rock phosphate) and potassium (K0, K1 and K2 received as KCl) as primary treatments, with plots further split into two levels of calcium application (Ca0 and Ca1 received as crushed limestone). Fertilization began three months after planting, occurring biannually (March/April and September/October) until the palms reached full production at approximately age three years, after which applications were reduced to annually in April. Complete nitrogen application history can be found in the paper by Oktarita *et al.* [41]. Agrochemicals were sprayed several times annually to control pests and weeds, primarily along the harvesting lines.

To isolate the effect of nitrogen (N) amendment, the three plots selected for this study received the same amounts of phosphorus (1 kg P palm^−1^ yr^−1^), potassium (1.5 kg K palm^−1^ yr^−1^) and no calcium. These fertilizer rates align with current industry standards. Each plot represented a different N treatment: N0 (control with no N), N1 (standard industry rate) and N2 (double the N1 rate). During this study, the palms were 2.8–3.3 years old. The N1 treatment received 51.5 and 103 kg N ha^−1^ yr^−1^ in the first and second fertilization events, respectively, while the N2 treatment received twice these rates.

### 2.3. Sampling design and soil flux measurement

To reflect common oil palm plantation practices, measurements were taken from two sampling positions: the CP position (where fertilizer was applied) and the farther-from-palm (FP) position (where no fertilizer was applied). The N0 treatment, although it did not receive N fertilizer, followed the same spatial stratification as N1 and N2 to account for potential root effects. The CP position was defined as a 1 m radius around each palm, covering an average of 6.2 m² (including the palm's basal area of approx. 0.5 m²). Given the triangular planting arrangement, the CP and FP positions accounted for 9 and 91% in each plot, respectively.

Soil respiration and CH_4_ fluxes were measured monthly from October 2012 to September 2013. However, owing to equipment failure, soil respiration measurements were not conducted in January and June 2013. Sampling frequency was increased following the two N fertilization events that took place on 23 October 2012 and 18 April 2013. During these events, gas samples were collected one day before fertilization (d−1), on the day of fertilization (d), daily for one week afterward (d+1 to d+7) and then every two days until d+29. This resulted in 19 sampling days over a 30 day fertilization period for each intensive sampling campaign.

During fertilization, urea was evenly distributed by hand within the chambers and collars located at the CP position in the N1 and N2 treatments. The applied fertilization rate was determined based on the surface ratio between the chamber or collar and the CP area, multiplied by the application rate. In the first fertilization event, the fertilizer was applied as granules, whereas in the second event, the granules were dissolved in distilled water before application to reduce nitrogen volatilization. Following standard management practices, urea was also homogeneously applied by hand within a 1 m radius around palm trunks outside the chambers and collars.

Soil CH_4_ fluxes were measured using the static chamber technique [42] in opaque white PVC chambers (26 cm in diameter and around 30 cm in height) permanently installed 2–3 cm into the soil. Chamber headspace was calculated at each sampling date using four height measurements taken between the soil surface and the top of the chamber. The chambers were deployed one month before data collection began. Each N treatment plot hosted 10 chambers: 5 replicates at the CP position and 5 replicates at the FP position. At the CP position, chambers were placed 50 cm from the palm trunk, while at the FP position, they were positioned midway between two palms outside harvesting paths or other management areas, following the design of Aini *et al.* [43]. Prior to sampling, the chambers were gently manually fanned for 30 s to ensure air mixing and then sealed with a vented lid featuring a centre port for gas sampling. Gas samples were collected from the enclosed chamber headspace immediately after closure and again at 10, 20 and 30 min thereafter. The samples were taken using a 50 ml syringe and transferred to 40 ml pre-evacuated glass vials. They were transported by road to the CIFOR laboratory in Jambi city and analysed by gas chromatography within a month. Samples were analysed using a Shimadzu 14A gas chromatograph equipped with a flame ionization detector for CH_4_ analysis [44]. A Porapak Q column was used for gas separation, and calibration was performed before each sequence of gas analysis with ultra-high-purity standard gases (1500, 2000, 10 000 and 40 000 ppb CH_4_). In most cases, CH_4_ concentration changes upon chamber closure followed a linear trend, allowing fluxes to be calculated via linear regression of the four collected samples, as detailed below. Whenever a sample had leaked or if the accumulation curve became nonlinear, the regression was based on three points instead (see further details in supplementary material, §S2):

(2.1) $$F_{{CH}_{4}}=\Delta {CH}_{4}\times 10^{-5}\times P\times H\times M\times 1440/R\;(T+273.15),$$

where $F_{{CH}_{4}}$ is the CH₄ flux rate (g CH_4_-C ha^−1^ d^−1^), ΔCH_4_ is the CH_4_ concentration change over time (ppb min^−1^), *P* is the air pressure within the chamber (Pa), *H* is the chamber height (m), *M* is the mass of C in CH₄ (12 g C mol^−1^ CH₄), 1440 is the time conversion factor (min d^−1^), *R* is the universal gas constant (8.314 Pa m^3^ mol^−1^ K^−1^), and *T* is the air temperature (°C).

Soil respiration was measured using dynamic closed chambers [45] connected to a portable infrared gas analyser. During the first six months (until March 2013), the same chambers used for CH_4_ flux measurements were connected to a LI-COR analyser (LI-840A, LI-COR Biosciences, USA) for CO_2_ concentration readings. Afterwards, smaller collars (10 cm in diameter and 5 cm in height) were installed and connected to a soil respiration chamber (SRC-1, PP Systems, UK) and an EGM-4 analyser (Environmental Gas Monitor, PP Systems, UK) for CO_2_ flux measurement. The collars were placed inside the chambers previously used a month before starting the monitoring. Soil respiration rates measured in March and in April before fertilizing were similar (*W* = 861.5, *p* = 0.210, *n*_1_ = 27, *n*_2_ = 30), indicating comparable flux readings between the LI-COR and EGM-4 analysers. Prior to closure, chambers or collars were gently manually fanned to homogenize headspace air. Following closure, CO_2_ concentrations were recorded automatically at 1 s (LI-COR) and 4.8 s (EGM-4) intervals for approximately 3 min, and fluxes were derived from the linear increase in CO_2_ concentration over time as follows:

(2.2) $$F_{CO_{2}}=\Delta CO_{2}\times 10^{-5}\times P\times H\times M\times 86400/R\;(T+273.15),$$

where $F_{CO_{2}}$ is the CO_2_ flux rate (kg CO_2_-C ha^−1^ d^−1^), ΔCO_2_ is the CO_2_ concentration change over time (ppm s^−1^), *P* is the air pressure within the chamber (Pa), *H* is the chamber height (m), *M* is the mass of C in CO_2_ (12 g C mol^−1^ CO_2_), 86 400 is the time conversion factor (sec d^−1^), *R* is the universal gas constant (8.314 Pa m^3^ mol^−1^ K^−1^), and *T* is the air temperature (°C).

Field GHG flux monitoring was conducted between 9 am and 2 pm. Previous research at the site [19] found no diel variation in soil CH_4_ fluxes. Additional analyses of soil respiration indicated no significant diel variation during relatively dry periods (less than 100 mm month^−1^), although higher CO₂ fluxes near palm bases were observed around 2 pm during wetter periods (more than 150 mm month^−1^) ([46] submitted). To minimize any potential bias associated with time-of-day effects, the order in which the chambers and collars were sampled was randomized.

Flux calculations for the CP and FP positions were based on the average of the five replicate chambers, with data recorded either monthly or daily during post-fertilization periods. Annual fluxes were estimated using a standard linear interpolation approach [47]. Briefly, the mean of two consecutive flux measurements (kg or g C ha^−1^ d⁻¹) was multiplied by the number of days between sampling dates to yield an interpolated flux (g or kg C ha^−1^) for that interval. Cumulative annual fluxes (Mg or kg C ha^−1^ yr⁻¹) were then obtained by summing all interpolated interval fluxes across the year. Sampling intervals averaged 28–30 days during non-fertilization periods and were shortened to approximately two days during and immediately following fertilization events to capture flux peaks.

### 2.4. Environmental variables and soil properties

Daily rainfall and air temperature were recorded using a rain gauge equipped with a thermometer (Delta OHM, v.HD2013-R) installed approximately 3 km away from the experimental site. Air pressure in the chambers was measured using a digital barometer (Greisinger electronic, Germany). Air temperature inside the chambers and soil temperature at a 10 cm depth outside each chamber were monitored using a digital thermometer (Greisinger electronic, GTH 1170).

Soil moisture and WT level were monitored independently of spatial position, following the observations of Hergoualc'h *et al.* [48], who reported homogeneous values across spatial positions in a young oil palm plantation on peat in Central Kalimantan. To determine soil moisture, one to five composite soil samples were collected monthly from the top 5 cm in each plot by mixing three individual soil samples taken with a knife. However, in October, June and August, 3–10 non-composite soil sample replicates per plot were collected using a metallic ring (8.4 cm diameter and 5 cm height). Samples were oven-dried at 60°C for a week, and the water content was determined based on a dry mass. The soil WFPS was calculated according to the formula by Linn & Doran [27] as the product of gravimetric soil moisture and bulk density (BD) divided by the porosity, assuming a peat particle density of 1.4 g cm^−3^. This default value is the average reported by Driessen & Rochimah [49] for lowland ombrogenous tropical peat, where BDs range between 0.1 and 0.25 g cm^−3^. The calculation used the plot-average BD without distinguishing between the two spatial positions (CP and FP). The WT level was monitored monthly alongside flux measurements in two perforated PVC wells (5 cm diameter and 2 m long) permanently and randomly placed inside each treatment plot.

At the end of the flux measurement period, six samples were collected from the soil top 5 cm in each treatment for chemical and physical analysis, with three samples taken from the CP position and three from the FP position. The soil was sampled using a metal ring with a 277 cm^3^ internal volume and oven-dried at 60°C for a week. The pH was measured in water (1:1 soil:water ratio). Total C and N concentrations were analysed by dry combustion with an elemental analyser (LECO TrusPec, St. Joseph, MI, USA). Available calcium was extracted using the Mehlich III solution, suitable for acidic soils, and quantified using inductively coupled plasma optical emission spectroscopy.

For root density determination, one soil sample was collected from the location of each chamber at the end of the experiment using a metallic ring (8.4 cm diameter and 5 cm height). The roots were separated from the soil by carefully rinsing in distilled water. Live and dead roots were separated, oven-dried at 60°C for 48 h and weighed. Root density was calculated as the total dry mass of live and dead roots per sample volume. This sampling depth was chosen to match the depth at which soil moisture, BD and chemical properties were characterized, ensuring consistency across all variables, and because fine root activity and microbial processes driving surface gas exchange are primarily concentrated in the aerobic uppermost peat layer. The peat BD was determined from five replicate core samples at each spatial position within each treatment plot. Cores were collected using a metallic ring (of the same dimension as previously mentioned) on three dates (June, July and August). The BD was calculated by dividing the soil dry mass by the ring volume.

Laboratory incubations were performed to assess N availability. Soil samples were collected to a 5 cm depth in each treatment in February 2013 when no fertilizer was applied and in April 2013 one day after fertilizer application. The samples were taken adjacent to (no fertilization) or inside (fertilization) each chamber using a ring sampler, as previously described. They were then transported to the laboratory and refrigerated at 4°C until incubation. Coarse roots and litter were manually removed from the samples before incubation. We followed the procedure by Hart *et al.* [50] to measure net mineralization and net nitrification rates. For each sample, a 10 g soil subsample was extracted in 100 ml of 2M KCl to determine inorganic N contents. The extracts were shaken for 1 h on a rotary shaker and allowed to settle for 24 h. A 20 ml aliquot of the supernatant was filtered (Whatman filter paper no. 42) and frozen for later analysis; NH_4_^+^ content was analysed on an autoanalyser (Bran+Luebbe^TM^) using the indophenol blue colorimetric method [51]. Determination of NO_3_^−^ was performed on a U-2001 spectrophotometer (Hitachi) using the brucine procedure [52]. Results from all extractions were corrected by subtracting the value of a blank.

A second 10 g subsample was incubated in the dark at room temperature (25–28°C) for 10 days. After this incubation period, the sample was extracted according to the procedures previously described. The net N mineralization rate was calculated as the change in inorganic N (NH_4_^+^ + NO_3_^−^) concentration; the net nitrification rate as the change in NO_3_^−^ concentration over 10 days. Initial inorganic N stocks were calculated from the first extraction, and all results are expressed in N unit on a per dry mass soil basis.

### 2.5. Statistics

The statistical analysis was performed using the InfoStat software [53], applying a significance level of 5% to evaluate the effects. To assess the adequacy of the analytical model, the normality of the residual distributions was evaluated using the Shapiro–Wilk test. The *t*-test and the non-parametric test of Wilcoxon were used to compare two means for normally and non-normally distributed variables, respectively. For multi-comparison, ANOVA and the non-parametric test of Kruskal–Wallis were performed, respectively, on normally and non-normally distributed variables. Annual cumulative soil respiration and CH_4_ emissions were considered significantly different between treatments or spatial positions when their 95% confidence intervals (CI) did not overlap.

Relationships between soil respiration or CH_4_ flux rates and environmental variables were examined using simple and multiple regression analyses. For analysis involving repeatedly monitored environmental variables (e.g. temperature, WT level and WFPS), chamber-level flux measurements and associated environmental variables were averaged separately for each plot and spatial position (CP and FP) on each sampling date. Consequently, each observation used in the regression analyses represented the mean value for a given plot × spatial-position × sampling-date combination. For analysis involving soil mineral N content, net N mineralization and net nitrification, which were measured only twice during the study (once during the outside-of-fertilization period and once during the April post-fertilization period), the same plot × spatial-position aggregation level was maintained. For the outside-of-fertilization period, soil fluxes and environmental variables measured on the same sampling date were used. For the April post-fertilization period, mean soil fluxes and environmental variables for each plot × spatial-position combination were calculated over the post-fertilization monitoring period and related to the corresponding measurements of soil mineral N content, net N mineralization and net nitrification.

To identify the environmental variables most strongly associated with soil fluxes, stepwise linear multiple regression was first performed using the full set of measured environmental variables. Variables retained in the final models were subsequently examined individually using simple linear and nonlinear regression analyses. Relationships were inspected across the full monitoring year and separately for three periods: the outside-of-fertilization period and the post-fertilization periods following the October and April fertilizer applications.

To further explore patterns in the data, relationships were also evaluated using daily averages binned into classes of selected environmental variables (e.g. 10 cm WT intervals and 10% WFPS intervals). Furthermore, relationships between cumulative annual soil respiration or CH_4_ emission rates and soil properties (table 1), as well as cumulative annual soil N_2_O emissions reported previously by Oktarita *et al.* [41], were examined using regression analyses.

**Table 1.** Soil (top 5 cm) properties, environmental parameters and soil N_2_O emissions in the N0, N1 and N2 treatments of the oil palm plantation fertilization trial detailed by spatial position (CP: close to palm and FP: farther from palm).

	N0	N1	N2
	**CP**		
pH	3.8 ± 0.1	4.1 ± 0.2	4.2 ± 0.1
C (%)	59.4 ± 0.2	56.6 ± 3.0	58.5 ± 1.5
N (%)	1.6 ± 0.1	1.5 ± 0.3	2.1 ± 0.0
C:N	36.9 ± 2.0	38.7 ± 5.7	28.3 ± 0.6
Ca (mg kg^−1^)	576.3 ± 157.6	845 ± 227.5	941.7 ± 118.1
bulk density (g d.m. cm^−3^)	0.21 ± 0.01	0.21 ± 0.01	0.21 ± 0.01
root density (Mg d.m. ha^−1^)	1.6 ± 0.5	2.6 ± 0.6	2.4 ± 1.3
soil temperature (°C)	27.9 ± 0.1^b α^	27.5 ± 0.1^a α^	28.3 ± 0.1^c α^
soil N_2_O emissions (kg N ha^−1^ yr^−1^)	5.7 ± 2.0 ^a α^	4.8 ± 0.9 ^a α^	19.3 ± 3.8 ^b^
	**FP**		
pH	3.8 ± 0.1	4.0 ± 0.2	4.0 ± 0.3
C (%)	59.3 ± 0.8^a^	60.6 ± 0.2^ab^	61.4 ± 0.5^b^
N (%)	1.7 ± 0.1	1.8 ± 0.2	2.0 ± 0.4
C:N	35.9 ± 2.1	34.0 ± 3.0	33.5 ± 7.5
Ca (mg kg^−1^)	556.3 ± 179.7	734.3 ± 356.0	677.0 ± 176.9
bulk density (g d.m. cm^−3^)	0.23 ± 0.01^b^	0.19 ± 0.01^a^	0.19 ± 0.01^a^
root density (Mg d.m. ha^−1^)	1.0 ± 0.7	1.1 ± 0.6	1.0 ± 0.4
soil temperature (°C)	28.3 ± 0.1^a β^	28.3 ± 0.1^a β^	28.8 ± 0.1^b β^
soil N_2_O emissions (kg N ha^−1^ yr^−1^)	23.7 ± 6.2 ^b β^	13.6 ± 2.9 ^a β^	27.3 ± 6.3 ^b^
	**CP and FP merged**		
WFPS (%)	63.5 ± 2.2^b^	57.7 ± 2.4^ab^	53.8 ± 2.2^a^
WT level (cm)	−49.8 ± 1.6^ab^	−47.7 ± 1.9^b^	−55.0 ± 1.7^a^

*Notes:* Letters (a, b and c) indicate a significant difference between N treatments, within a spatial position. Symbols (α and β) indicate a significant difference between spatial positions, within an N treatment. In the absence of a significant difference, no letters are displayed; *n* = 3 for all soil properties except for bulk density (*n* = 15). Soil averages of mineral N content, mineralization and nitrification rates were based on five samples, and root density averages on five samples. Soil annual N_2_O emissions were cumulated over 47 sampling days [41]; *n* is around 231 for soil temperature, 90 for WFPS and 67 for WT level. Values are mean/cumulative values ± standard error (SE).

## 3. Results

### 3.1. Soil properties and environmental variables

The soil was acidic with pH values around 4 and had high C and N concentrations (table 1) as compared to global peatland averages [54]. The N2 treatment presented a greater C concentration than the N0 treatment at the FP position (df = 2, *H* = 6.49, *p* = 0.009). In addition, the soil N concentration and C:N ratio were homogeneous among treatments. Calcium levels were low [55] and similar across treatments. The BD was higher in the N0 treatment than the others at the FP position (df = 2, *F* = 4.13, *p* = 0.023). Finally, roots were equally dense across treatments and spatial positions, with live roots accounting for an average of 80% of the total roots.

In all treatments, inorganic N pools were dominated by NH_4_⁺, with considerably greater concentrations than NO_3_^−^ (*W* = 577, *p* < 0.0001, *n*_1_ = 20, *n*_2_ = 20 for N0; *W* = 549, *p* < 0.0001, *n*_1_ = 19, *n*_2_ = 19 for N1; *W* = 509.5, *p* = 0.007, *n*_1_ = 20, *n*_2_ = 20 for N2), consistently across both spatial positions (*W* = 1182, *p* < 0.0001, *n*_1_ = 29, *n*_2_ = 29 for CP; *W* = 1242, *p* < 0.0001, *n*_1_ = 30, *n*_2_ = 30 for FP) and during both observation periods (*W* = 1312, *p* < 0.0001, *n*_1_ = 30, *n*_2_ = 30 for February without fertilization and *W* = 1201, *p* < 0.0001, *n*_1_ = 29, *n*_2_ = 29 for April after fertilization) (supplementary material §S3, supplementary material table S3). Net mineralization rates exceeded nitrification rates (*W* = 3914, *p* = 0.030, *n*_1_ = 59, *n*_2_ = 59). In the absence of fertilizer application, soil NH_4_^+^ contents were high overall but lower in the N2 than the N1 treatment at both spatial positions (df = 2, H = 7.5, *p* = 0.024 for CP; df = 2, *H* = 9.5, *p* = 0.009 for FP). Following fertilization, NH_4_⁺ levels were higher in the N2 than the N0 treatment at the CP position (df = 2, *H* = 7.9, *p* = 0.019), while no treatment differences were observed at the FP position. Within the N2 treatment, post-fertilization NH_4_^+^ contents were higher at the CP than the FP position (*W* = 38, *p* = 0.032, *n*_1_ = 5, *n*_2_ = 5). Soil NO_3_^−^ contents were low and uniform among treatments (df = 3, *H* = 2, *p* = 0.367 for CP; df = 2, *H* = 3, *p* = 0.219 for FP). They were consistently higher under fertilizer application than in its absence (*W* = 38, *p* = 0.032 for N0 CP; *W* = 40, *p* = 0.008 for N0 FP; *W* = 30, *p* = 0.016 for N1 CP; *W* = 40, *p* = 0.008 for N1 FP; *W* = 40, *p* = 0.008 for N2 CP; *W* = 40, *p* = 0.008 for N2 FP; *n*_1_ = 5, *n*_2_ = 5 except for N1 CP where *n*_1_ = 4). Since this pattern was independent of spatial position and N treatment, it probably reflects a seasonal effect rather than a direct fertilization effect. In the absence of fertilizer, net mineralization rates were higher at the CP position than the FP position in the N2 treatment (*W* = 37, *p* = 0.048, *n*_1_ = 5, *n*_2_ = 5). After fertilization, they were higher in N2 than in N0 and N1 at the FP position (df = 2, *H* = 8.8, *p* = 0.012). Without fertilizer, nitrification rates differed between treatments only at the FP position, where values were lower in N2 compared to N1 (df = 2, *H* = 6.3, *p* = 0.042).

Complementary soil properties and soil inorganic N dynamics descriptions are available in the paper by [41].

**Figure 1.**
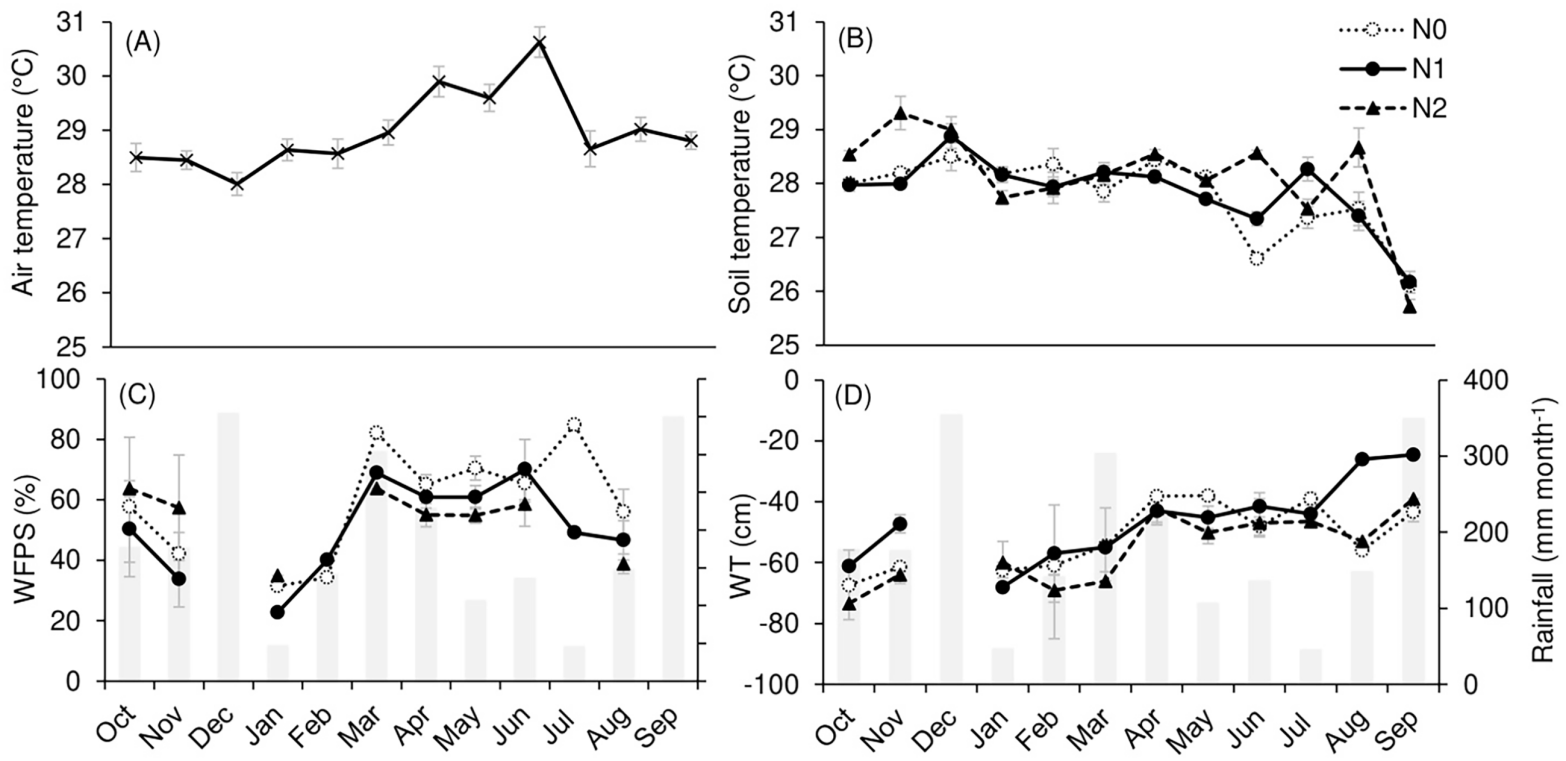
Average monthly air temperature (A), soil temperature (top 10 cm) (B), WFPS (top 5 cm) (C), water table level (WT) (D) and cumulative monthly rainfall (grey vertical bars) (C and D) in the N0, N1 and N2 treatments of the oil palm plantation trial. Error bars indicate standard errors.

Average monthly air temperatures ranged from 28.0 to 30.6°C (figure 1A), with higher values recorded between April and June compared to the rest of the year (*T* = −2.6, *p* = 0.01, *n*_1_ = 810, *n*_2_ = 567). Average soil temperatures remained stable at around 28°C, except in September when they dropped to 26°C (figure 1B). Across treatments, soil temperatures were lower at the CP position than the FP position (*T* = −5, *p* < 0.0001, *n*_1_ = 231, *n*_2_ = 230 for N0; *T* = −9.7, *p* < 0.0001, *n*_1_ = 231, *n*_2_ = 233 for N1; *T* = −3.3, *p* = 0.0012, *n*_1_ = 231, *n*_2_ = 231 for N2) (table 1). Rainfall did not follow a clear seasonal pattern during the monitoring period (figure 1C). There were two dry months (January and July) with less than 50 mm of cumulative rainfall per month and two very wet months (December and September) with about 350 mm of rainfall per month.

The soil average WFPS across treatments amounted to just under 60%, indicating optimal conditions for microbial activity [27]. The WFPS reached its lowest levels during and after the driest month of January, increased when rainfall resumed in March and then remained relatively stable (figure 1C). On average, the WFPS was significantly lower in the N2 treatment than in the N0 treatment (df = 2, *H* = 11.6, *p* = 0.003) (table 1). The WT level ranged from −24.5 to −73.4 cm (figure 1D), with an average of −50.8 ± 1.0 cm across treatments. It was lower in N2 compared to N1 (df = 2, *H* = 6.7, *p* = 0.04).

### 3.2. Soil greenhouse gas fluxes

Both soil respiration and CH_4_ fluxes are presented first outside the fertilization periods, second during the 29 days following the October (23/10–21/11/2012) and April (18/4–15/5/2013) fertilizations and third on an annual scale.

#### 3.2.1. Soil respiration outside fertilization periods

**Figure 2.**
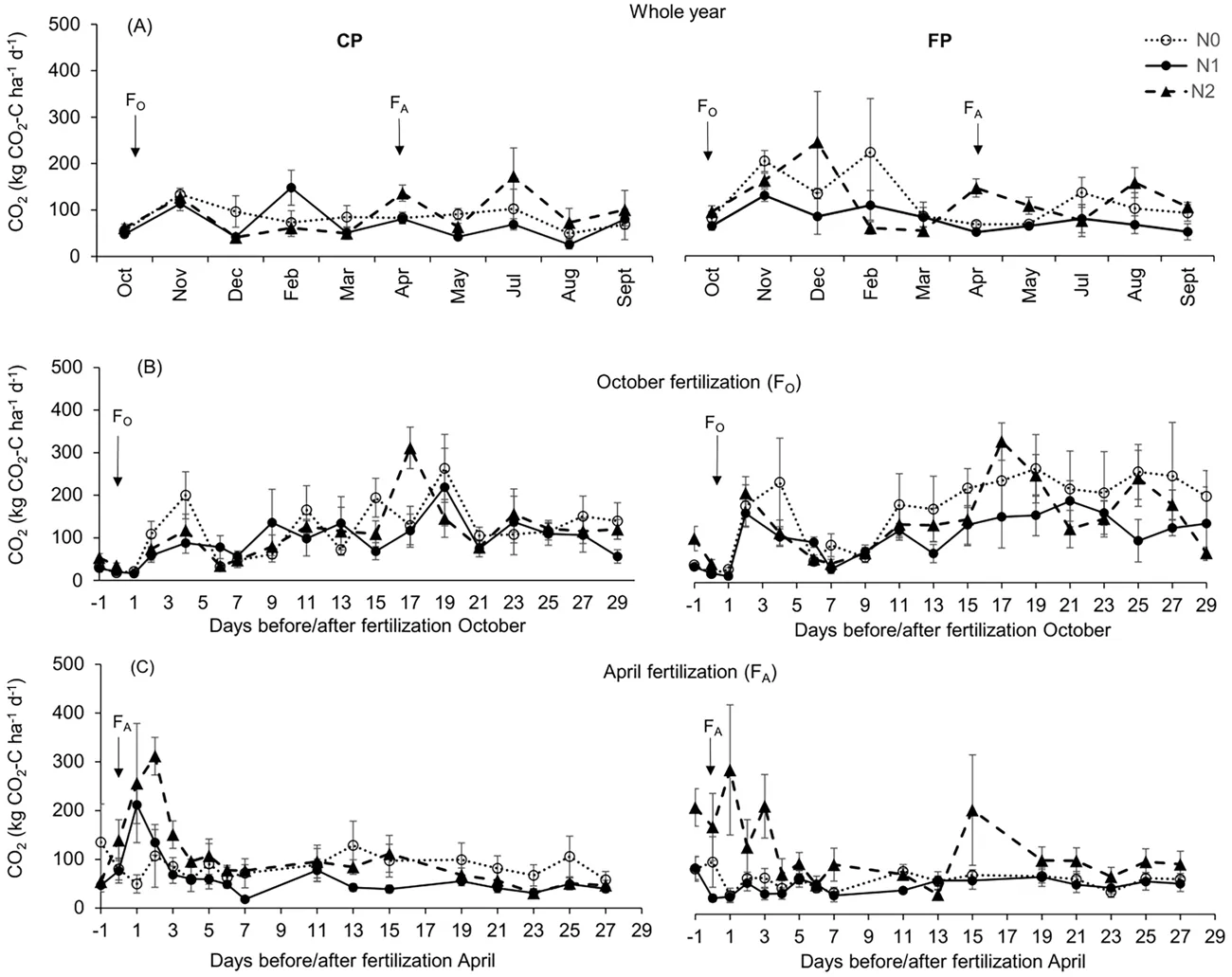
Average soil respiration over the year (A) and after the fertilizations of October (B) and April (C) at the close-to-palm (CP) and farther-from-palm (FP) positions of the N0, N1 and N2 treatments of the oil palm plantation fertilization trial. The arrows indicate fertilization events; error bars indicate standard errors.

Monthly soil respiration was highly spatio-temporally variable, with lowest and highest average rates (kg CO_2_-C ha^−1^ d^−1^) of 25.7 ± 9.4 (N1 CP in August) and 248.7 ± 110.8 (N2 FP in December) (figure 2A). The N0 and N2 treatments displayed higher soil respiration rates than the N1 treatment at the FP position (df = 2, *H* = 7.2, *p* = 0.028), while no treatment effect was observed at the CP position (table 2). A spatial effect was noticeable in the N0 and N2 treatments where soil respiration was substantially higher at the FP position than at the CP one (*W* = 2295, *p* = 0.010, *n*_1_ = 45, *n*_2_ = 44 for N0; *W* = 2317, *p* = 0.006, *n*_1_ = 45, *n*_2_ = 44 for N2). On average, soil respiration was lower outside the fertilization periods compared to the two post-fertilization periods in the N2 treatment at the CP position (df = 2, *H* = 6.8, *p* = 0.034), indicating a clear fertilizer effect.

**Table 2.** Average and cumulative annual soil respiration ± SE outside the fertilization periods (307 days) and during the two post-fertilization periods (29 days each) in the N treatments (N0, N1 and N2) of the oil palm plantation fertilization trial.

		average soil respiration rate (kg CO_2_-C ha^−1^ d^−1^)			annual soil respiration rate (Mg CO_2_-C ha^−1^ yr^−1^)	
	spatial position	outside fertilization	post-fertilization October	post-fertilization April	per spatial position	plot scale
N0	CP	76.0 ± 11.9 ^α A^	108.4 ± 10.3 ^B^	83.2 ± 8.2 ^a AB^	20.5 ± 3.3	27.3 ± 2.8
					[14.0–26.9]	[21.8–32.8]
	FP	109.9 ± 15.9 ^b β B^	166.4 ± 17.5 ^B^	67.8 ± 4.5 ^a A^	28.0 ± 3.1	
					[22.0–34.0]	
N1	CP	58.5 ± 7.4	93.2 ± 10.9	65.8 ± 6.3 ^a^	13.6 ± 1.1	18.8 ± 2.5
					[11.5–15.8]	[14.0–23.7]
	FP	73.3 ± 8.2 ^a A^	109.4 ± 9.9 ^B^	55.6 ± 3.6 ^a A^	19.4 ± 2.7	
					[14.0–24.7]	
N2	CP	75.2 ± 10.4 ^α A^	106.5 ± 9.3 ^αB^	110.3 ± 12.1 ^b B^	20.7 ± 3.1	28.5 ± 3.0
					[14.7–26.7]	[22.7–34.4]
	FP	123.5 ± 16.9 ^b β^	142.9 ± 12.4 ^β^	127.2 ± 14.6 ^b^	29.3 ± 3.3	
					[22.9–35.8]	

*Notes:* Letters (a, b and c) indicate a significant difference between N treatments, within a spatial position and a period. Symbols (α and β) indicate a significant difference between spatial positions, within an N treatment and a period. Letters (A, B and C) indicate a significant difference between periods, within a spatial position of an N treatment. In the absence of a significant difference, no letters are displayed. *n* is about 55 outside fertilization periods and 90 during post-fertilization periods. Cumulative emission rates are based on 47 sampling days. Emission rates are presented for each spatial position (CP: close to palm and FP: farther from palm) and at the plot scale (i.e. 91% CO_2 FP_ + 9% CO_2 CP_). Overall, 95% CI of cumulative annual emissions are presented in brackets.

#### 3.2.2. Post-fertilization soil respiration

During the period following the October fertilization, soil respiration fluctuated greatly with no clear fertilizer effect at the CP position of the N1 and N2 treatments, where the fertilizer was placed (figure 2B). All treatments had similar soil respiration rates at both spatial positions (table 2). Moreover, in the N2 treatment, soil respiration was higher at the FP position than the CP position (*W* = 6232, *p* = 0.035, *n*_1_ = 78, *n*_2_ = 74).

**Figure 3.**
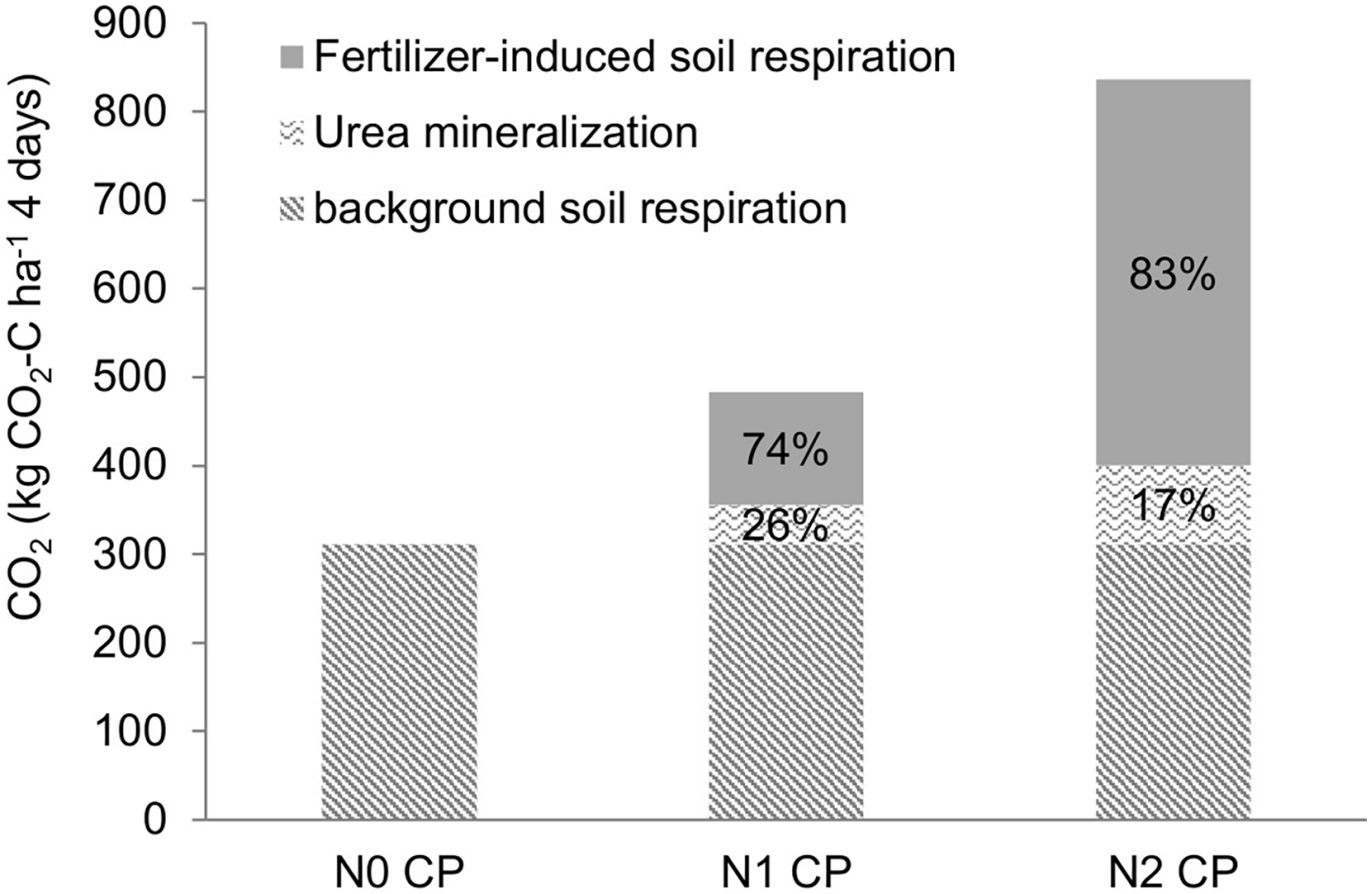
Partitioning of April's post-fertilization cumulative (days 0–4) soil respiration at the CP position in the oil palm plantation treatments (N0, N1 and N2). Background respiration in N1 and N2 was assumed to equal background respiration in N0. The carbon in the applied urea (45 and 89 kg C ha^−1^ in N1 and N2) was assumed to have fully volatilized as CO_2_ [56]. The remaining emissions correspond to fertilizer-induced respiration. The percentages indicate the share of urea mineralization and fertilizer-induced respiration contributing to emission surplus resulting from fertilization.

After fertilizer application in April, soil respiration at the CP position peaked on day 1 in the N1 treatment and on days 1 and 2 in the N2 treatment before returning to levels similar to those in the N0 treatment on day 4 (figure 2C). In N1 and N2, emissions cumulated over the four days following N application were higher than in N0 with an emission surplus resulting from urea mineralization (17–26%) and fertilizer-induced respiration (74–83%) (figure 3). Further, the higher soil respiration in N2 compared to N0 and N1 at the CP position strongly suggests a fertilizer effect in this treatment over 29 days post-fertilization (df = 2, *H* = 13.2, *p* = 0.001) (table 2). Some peaks were also observed at the FP position of the N2 treatment, particularly on days 1, 3 and 15. Consequently, soil respiration at the FP position was also greater in N2 than in the other treatments (df = 2, *H* = 26.3, *p* < 0.0001). There was no spatial effect in either treatment, with similar average rates at the CP and FP positions (*W* = 6189, *p* = 0.925, *n*_1_ = 79, *n*_2_ = 78 for N0; *W* = 6563, *p* = 0.675, *n*_1_ = 80, *n*_2_ = 80 for N1; *W* = 6443, *p* = 0.325, *n*_1_ = 79, *n*_2_ = 78 for N2) (table 2). Over this period, daily fluctuations in soil respiration were partially positively linked to soil temperature ( table 3, eq. 1). A more robust relationship was found between monthly soil respiration rate and soil net nitrification rate (table 3, eq. 2).

**Table 3.** Relationships between soil respiration rates and environmental variables per period (NF: outside fertilization periods and *F_A_*: post-fertilization of April) and across periods.

model	*R*^2^	*n*	eq.
per period			
CO_2_ *_FA_* = 22.4^**^ (6.7) × Soil *T* − 545.3^**^ (187.5)	0.11	90	(1)^a^
CO_2_ *_FA_* = 16.1^*^ (4.4) × Nit + 64.0^**^ (8.5)	0.77	6	(2)^b^
across periods			
CO_2_ = −1.9^***^ (0.4) × WT + 21.9^***^ (5.0) × Soil *T* − 0.4^*^ (0.2) × WFPS − 590.8^***^ (141.0)	0.33	112	(3)^a^
CO_2_ = −0.8^*^ (0.3) × WFPS + 139.5^**^ (17.4)	0.51	9	(4)^c^
CO_2_ = 87.2^*^ (27.6) × Soil *T* − 191.8^*^ (77.1) × NO_3_^−^/(NO_3_^−^ + NH_4_^+^) − 2314.6^*^ (774.0)	0.62	12	(5)^b^
cumulative annual			
CO_2_ = 11.3^*^ (3.5) × Soil *T* − 295.7^*^ (98.8)	0.72	6	(6)
CO_2_ = 0.6^*^ (0.2) × N_2_O + 13.2^**^ (2.6)	0.78	6	(7)
CO_2_ = 0.5^**^ (0.06) × N_2_O − 0.02^*^ (0.004) × Ca + 26.2^**^ (3.1)	0.97	6	(8)

*Notes:* The models are presented with slope (SE), intercept (SE) and level of significance: * *p* < 0.05, ** *p* < 0.01 and *** *p* < 0.001. Soil CO_2_ rates are expressed in kg CO_2_-C ha^−1^ d^−1^ or Mg CO_2_-C ha^−1^ yr^−1^ (for cumulative annual), soil temperature (soil *T*) is in °C, soil net nitrification rate (Nit) in mg N kg^−1^ d.m. d^−1^, water table level (WT) in cm, soil water-filled pore space (WFPS) in %, soil NO_3_^−^ and NH_4_^+^ content in mg N kg^−1^ d.m., soil N_2_O emissions in kg N ha^−1^ yr^−1^ and soil calcium content (Ca) in mg kg^−1^.

^a^ Daily averages.

^b^ Monthly averages.

^c^ Daily averages binned into 10% WFPS intervals (from [10; 20] to [90; 100]).

#### 3.2.3. Annual soil respiration and relationships with other variables across periods

Annual soil respiration varied between treatments and spatial positions, tending to be higher in the N0 and N2 treatments than in N1, and at the FP position than at the CP position across all treatments, though none of these differences were significant (table 2).

Across periods, daily soil respiration increased with a deeper WT below the surface, higher soil temperature and lower soil WFPS (table 3, eq. 3). A relationship with a higher explanatory power was also found between daily soil respiration and the WFPS alone (table 3, eq. 4), while the WT alone did not explain temporal variations in soil respiration (*R*^2^ = 0.070). Moreover, monthly soil respiration was related to both soil temperature (positively) and soil mineral nitrogen content (negatively) (table 3, eq. 5). On an annual scale, soil respiration was positively related to average soil temperature (table 3, eq. 6) and annual soil N_2_O emissions (table 3, eq. 7). This last relationship had a higher explanatory factor when including soil calcium content (table 3, eq. 8).

#### 3.2.4. Soil CH_4_ emissions outside fertilization periods

**Figure 4.**
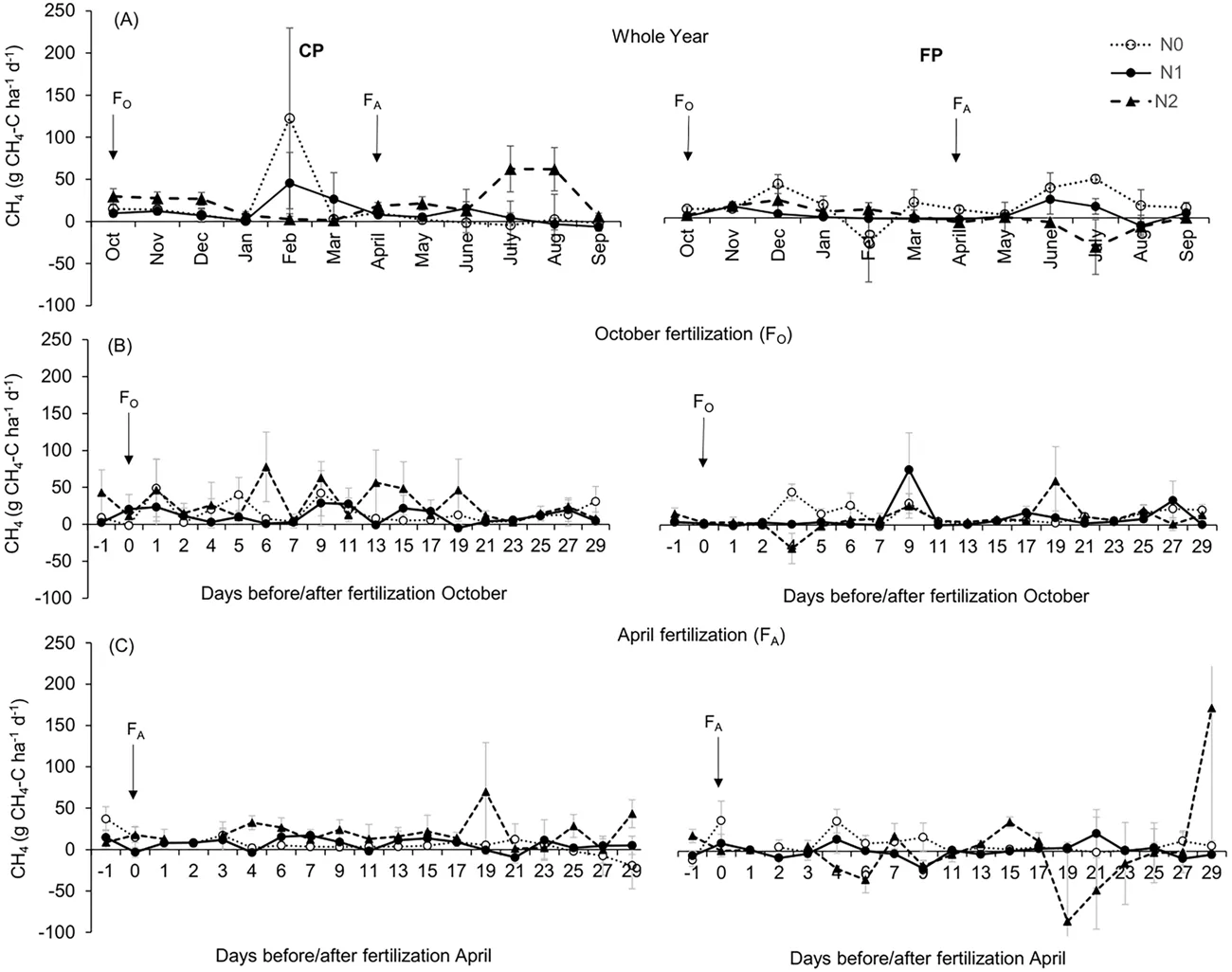
Average soil methane fluxes over the year (A) and after the fertilizations of October (B) and April (C) at the close-to-palm (CP) and farther-from-palm (FP) positions of the N0, N1 and N2 treatments of the oil palm plantation fertilization trial. The arrows indicate fertilization events; error bars indicate standard errors.

Monthly average soil CH_4_ fluxes (g CH_4_-C ha^−1^ d^−1^) ranged from a minimum of −35.8 ± 33.7 (N2 FP in July) to a maximum of 122.4 ± 107.3 (N0 CP in February) ( figure 4A), with very high spatial and temporal variability. The N2 treatment exhibited higher CH_4_ emissions than the N1 treatment at the CP position (df = 2, *H* = 9.9, *p* = 0.007) (table 4). At the FP position, the N0 treatment had higher emissions than the N1 treatment (df = 2, *H* = 11.5, *p* = 0.003). No spatial effect on CH_4_ emissions was detected (*W* = 27 031, *p* = 0.65, *n*_1_ = 163, *n*_2_ = 163). Further, soil emissions of CH_4_ in this period were similar to emissions in post-fertilization periods, in all treatments (df = 3, *H* = 9.4, *p* = 0.009).

**Table 4.** Average and cumulative annual methane emissions ± SE outside the fertilization periods (307 days) and during the two post-fertilization periods (29 days each) in the N treatments (N0, N1 and N2) of the oil palm plantation fertilization trial.

		average emission rate (g CH_4_-C ha^−1^ d^−1^)			annual emission rate (kg CH_4_-C ha^−1^ yr^−1^)	
	spatial position	outside fertilization	post-fertilization October	post-fertilization April	per spatial position	plot scale
N0	CP	16.1 ± 10.8 ^ab^	15.5 ± 3.6 ^b^	4.3 ± 2.5 ^a^	5.0 ± 3.2	3.9 ± 1.5
					[−1.3–11.3]	[0.9–6.8]
	FP	14.0 ± 5.9 ^b^	11.5 ± 2.1 ^b^	8.1 ± 3.6 ^b^	3.8 ± 1.6	
					[0.6 – 6.9]	
N1	CP	11.2 ± 5.2 ^a^	11.8 ± 3.2 ^a^	6.1 ± 2.2 ^a β^	2.9 ± 1.4	0.7 ± 0.6
				[0.2–5.6]	[−0.5 to 1.9]
	FP	3.1 ± 2.3 ^a^	9.4 ± 3.5 ^a^	0.2 ± 2.2 ^a α^	0.5 ± 0.7	
					[−0.8 to 1.8]	
N2	CP	24.4 ± 5.7 ^b^	27.7 ± 5.9 ^b^	20.9 ± 4.9 ^b β^	7.1 ± 1.6 ^β^	1.0 ± 1.3
					[4.1–10.2]	[−1.6 to 3.6]
	FP	4.4 ± 4.2 ^ab^	8.2 ± 3.5 ^a^	−3.2 ± 10.5 ^ab α^	0.4 ± 1.5 ^α^	
					[−2.4 to 3.3]	

*Notes:* Letters (a, b and c) indicate a significant difference between N treatments, within a spatial position and a period. Symbols (α and β) indicate a significant difference between spatial positions, within an N treatment and a period. In the absence of a significant difference, no letters are displayed; *n* is about 55 outside the fertilization periods and 90 during post-fertilization periods. Cumulative emission rates are based on 47 sampling days. Emission rates are presented for each spatial position (CP: close to palm and FP: farther from palm) and at the plot scale (i.e. 91% CH_4 FP_ + 9% CH_4 CP_). Overall, 95% CI of cumulative annual emissions are presented in brackets.

#### 3.2.5 Post-fertilization CH_4_ emissions

Following the October fertilization, average soil CH_4_ emissions exhibited moderate fluctuations with the highest variations in the N2 treatment at the CP position and in the N1 treatment at the FP position (figure 4*B*). Both the N0 and N2 treatments showed higher emissions than the N1 treatment at the CP position (df = 2, *H* = 13.5, *p* = 0.001) (table 4). At the FP position, the N0 treatment had higher emissions than the other two treatments (df = 2, *H* = 11.1, *p* = 0.004). There was no consistent spatial effect as differences between the FP and CP positions were not significant in any of the treatments (*W* = 7896, *p* = 0.74, *n*_1_ = 90, *n*_2_ = 89 for N0; *W* = 8225, *p* = 0.54, *n*_1_ = 89, *n*_2_ = 90 for N1; *W* = 8374, *p* = 0.08, *n*_1_ = 88, *n*_2_ = 88 for N2) (table 4).

Throughout the post-fertilization period of April, average soil CH_4_ emissions remained relatively stable except in the N2 treatment. There, occasional spikes were observed at the CP position, while the FP position experienced one instance of high uptake and one of high emission (figure 4C). Emissions were significantly higher in the N2 treatment compared to the other treatments at the CP position (df = 2, *H* = 13.7, *p* = 0.001) (table 4), suggesting a slight fertilizer effect. They were higher in the N0 treatment than the N1 one at the FP position (df = 2, *H* = 9.56, *p* = 0.008). A spatial effect was observed in the N1 and N2 treatments (*W* = 7938, *p* = 0.008, *n*_1_ = 84, *n*_2_ = 84 for N1; *W* = 4839, *p* = 0.001, *n*_1_ = 78, *n*_2_ = 75 for N2) with higher emissions at the CP than the FP position. No such effect occurred in the N0 treatment (*W* = 6754, *p* = 0.430, *n*_1_ = 82, *n*_2_ = 80) (table 4).

#### 3.2.6. Annual CH_4_ emissions and relationships with other variables across periods

Annual soil CH_4_ emissions were relatively low across all treatments, ranging from 0.4 ± 1.5 to 7.1 ± 1.6 kg C ha^−1^ yr^−1^ (table 4). Although not significant, CH_4_ emission rates tended to be highest in the N2 treatment at the CP position, and in the N0 treatment at the FP position, the latter being approximately 90% higher than the other treatments. The only significant spatial difference, with greater emissions at the CP than the FP position, was found in the N2 treatment.

Plot-scale soil annual CH_4_ rates tended to be higher in the N0 treatment than the N1 and N2 treatments (around 80 and 70% higher, respectively), though these differences were not significant. The two fertilized treatments had similar emission rates.

Across periods, average soil CH_4_ fluxes increased with a deeper WT level below the surface (table 5, eq. 8). The WFPS, on the other hand, was not a significant control of soil CH_4_ flux variation (*R*^2^ = 0.3, *p* = 0.128). On an annual basis, soil CH_4_ emissions were higher as soil NO_3_^−^ contents (in the absence of fertilization) were lower (table 5, eq. 9).

**Table 5.** Relationship between soil CH_4_ rates and environmental variables across all periods.

model	*R*^2^	n	eq.
across periods			
CH_4_ = −0.37^***^ (0.05) × WT − 9.01^**^ (2.98)	0.91	7	(8)^a^
cumulative annual			
CH_4_ = −0.85^*^ (0.33) × NO_3_^−^ + 8.10^**^ (1.99)	0.63	6	(9)

*Notes:* The models are presented with slope (SE), intercept (SE) and level of significance: * *p* < 0.06, ** *p* < 0.05 and *** *p* < 0.001. CH_4_ rates are expressed in g CH_4_-C ha^−1^ day^−1^ or kg CH_4_-C ha^−1^ yr^−1^ (for cumulative annual), water table level (WT) in cm and soil NO_3_^−^ content in mg N kg^−1^ d.m. The latter are the averages measured outside the fertilization period.

^a^ Daily averages binned into 10 cm WT intervals (from [−20; −30] to [−80; −90]).

## 4. Discussion

### 4.1. Soil respiration

Annual soil respiration at the study site fell within the upper range of values reported for oil palm plantations on peat (supplementary material, §S4, supplementary material, fig. S4A). In the N0 and N2 treatments (27–28 Mg C ha^−1^ yr^−1^), rates were comparable to those measured by Comeau *et al.* [40] in a seven-year-old plot from the same plantation and by Swails *et al.* [23] and Manning *et al.* [36] in five- and eight-year-old plantations in Indonesia and Malaysia, respectively. The annual rate in the N1 treatment (19 Mg C ha^−1^ yr^−1^) closely matched that observed in a seven-year-old plantation in Malaysia by Melling *et al.* [57]. Across treatments, values stood at the higher end of the literature-derived relationship for oil palm plantations on peat, which shows annual soil respiration declining as soil C:N decreases (supplementary material, §S5, supplementary material, fig. S5). This pattern is consistent with findings from Swails *et al.* [58], who reported reduced soil heterotrophic respiration with lower C:N ratios over a 30-year rotation in a Kalimantan plantation. The higher C:N ratio at our site (34.6 ± 1.8, *n* = 3) relative to the literature (29.4 ± 1.0, *n* = 17) was driven by elevated C concentration (58.4 ± 1.0 vs. 51.7 ± 1.3), as N concentration was similar (1.8 ± 0.1 at all sites). In the same plantation, Comeau [59] documented a substantial volume of woody debris in the top 20 cm surface peat (12% of less than 15 cm^3^ small to greater than 500 cm^3^ large logs) and suggested that their decay probably contributes substantially to the high soil heterotrophic respiration observed.

Annual soil respiration tended to be greater in the N0 and N2 treatments than the N1 one, at both spatial positions and at the plot scale (table 2). Contributing factors included differences in soil temperature (table 3, eq. 6), soil N_2_O emissions alone (table 3, eq. 7) or in combination with calcium content (table 3, eq. 8) . The temperature dependence of soil respiration—both autotrophic and heterotrophic—is well established [60] and has been observed in other oil palm plantations on peat [16,48]. Because soil respiration drives soil organic matter mineralization, thereby increasing soil nitrogen availability, its positive relationship with soil N₂O emissions is mechanistically well-founded. Although soil calcium content did not differ significantly among treatments or positions, its spatial heterogeneity may have further influenced soil respiration. Low calcium contents are typical of ombrotrophic peatlands [55], and calcium application has been shown to reduce soil CO_2_ production by prompting mineral-associated soil organic matter and mediating coupled biotic–abiotic mechanisms [61]. Across treatments, annual soil respiration was higher at the FP position than CPs (table 2), contrary to patterns reported for oil palm plantations on peat [22,36,48,62] and to our research hypothesis. This finding calls into question the assumption made by several studies [63,64] that respiration measured near palms represents total soil respiration, while that measured farther away reflects only heterotrophic respiration, an assumption that holds only if heterotrophic respiration (Rh) is spatially consistent between positions. However, higher soil temperatures and N₂O emissions observed at FPs, coinciding with higher respiration (table 3, eqs. 6, 7), suggest that Rh may itself be greater at FP than CP, potentially driven by enhanced peat mineralization, which would further complicate the partitioning of soil respiration components. Dariah *et al.* [62] found only a weak influence of root density in spatial respiration patterns, consistent with this interpretation.

Following the April fertilization, soil respiration was greatly stimulated in both N1 and N2 treatments, with cumulative CP-position values over four days post-application exceeding those of the unfertilized treatment by 55 and 168%, respectively (figure 3). This priming effect was far more important than the 12% plot-scale increase over seven days post-fertilization reported by Comeau *et al.* [33], which was mainly owing to higher respiration at the FP position, potentially because of fertilizer leakage. The difference in response may be explained by the lower N rate applied by Comeau *et al.* [33] (35 kg N ha^−1^) as compared to rates in the present study (103 and 206 kg N ha^−1^ in N1 and N2). Most of the emission surplus in the amended treatments was fertilizer-induced (figure 3), with N boosting either root respiration, soil organic matter decomposition or both. The enhancement of soil respiration under increased N availability was clearly evidenced by its strong correlation with net nitrification (table 3, eq. 2). Nonetheless, the pronounced but short-lived response following the April fertilization did not translate into detectable differences in annual emissions among treatments (table 2) confirming our research hypothesis.

Temporal variations in soil respiration were linked to dynamics in soil temperature (table 3, eqs 1, 3 and 5), moisture (WT and WFPS, table 3, eqs. 3 and 4) and the proportion of nitrate in the soil inorganic nitrogen pool (NO_3_^−^/(NO_3_^−^ + NH_4_^+^), table 3, eq. 5). While soil temperature alone (table 3, eq. 1) explained little of the temporal variation in soil respiration (*R*^2^ = 0.11), its explanatory power increased when combined with changes in the soil inorganic nitrogen index (table 3, eq. 5). The observed increase in respiration rates as nitrate became less dominant in the mineral nitrogen pool aligns with the positive relationship between soil respiration and N_2_O emissions (table 3, eq. 8). The relationship, including WT and WFPS in addition to soil temperature, had low explanatory power (table 3, eq. 3, *R*^2^ = 0.33). The observed increase in soil respiration as the WT decreases further below the surface mirrors patterns reported in other oil palm plantations on peat [16,18,48]. In contrast, the WFPS alone accounted for about half of the temporal variability in soil respiration (table 3, eq.4), supporting our hypothesis that the WFPS exerts stronger control than the WT. This is probably because WFPS more directly captures the soil moisture conditions experienced by microbial communities at the peat surface. Furthermore, as ombrotrophic systems, these peatlands receive water input exclusively through precipitation, meaning the upper soil layers wet and dry from the surface downward rather than from the rising and falling WT below. Consequently, surface WFPS may decouple from WT dynamics, rendering WT a less sensitive proxy for the moisture environment governing microbial activity. The observed rise in soil respiration with decreasing WFPS—reflecting greater oxygenation of soil pores—has similarly been documented in other drained tropical peatland studies [34,65]. While our study assumed no spatial variation in soil WFPS owing to the young age of the palms, studies conducted in older plantations should consider spatially stratified soil moisture measurements, as higher moisture levels have been reported at the FP position compared to the CP position [19]. Finally, the regression analyses were intended to identify environmental factors associated with variation in soil respiration across treatments, spatial positions and monitoring periods. While the observed relationships are consistent with established controls on peat respiration, repeated observations through time may introduce some degree of temporal dependence among measurements. The reported relationships should, therefore, be interpreted as indicative of potential environmental drivers rather than definitive evidence of causal mechanisms.

### 4.2. Soil CH_4_ emissions

Annual soil CH_4_ emissions at the site were within the range of rates reported for oil palm plantations on peat (supplementary material, §S4, supplementary material fig. S4B). Emissions in the N0 treatment (3.9 kg C ha^−1^ yr^−1^) were close to those reported by Lau *et al.* [66] in a six-year-old plantation in Malaysia, while rates for the other treatments (0.7–1 kg C ha^−1^ yr^−1^) were comparable to values measured by Swails *et al.* [23] in a six-year-old plantation in Indonesia.

Annual soil CH_4_ emissions tended to be more important in N0 than in the fertilized treatments, both at the plot-scale and at the FP position (table 4). This pattern persisted consistently across all observation periods, regardless of fertilizer application. A spatial effect was observed in the fertilized treatments, with higher annual emissions at the CP position compared to the FP position (table 4). This effect became significant after the April fertilization and showed a similar, though non-significant, tendency under unfertilized conditions. Differences among treatments and spatial positions were strongly associated with soil NO_3_^−^ content outside the fertilization period (table 5, eq. 9; supplementary material, table S3). Similar reduced soil CH_4_ efflux under higher NO_3_^−^ concentrations has been reported in peatlands, where the high organic matter of the peat enhances nitrite-dependent anaerobic CH_4_ oxidation [67].

A transient fertilization effect was observed at the CP position of the N2 treatment 19 days after the April fertilizer application (figure 4*C*). The temporary increase in emissions was probably related to elevated NH_4_^+^ concentrations at that time, which can inhibit CH_4_ oxidation, as previously reported by Melling *et al.* [68] in a Malaysian oil palm plantation on peat. This emission peak also coincided with a pronounced N_2_O release [41]. In contrast, no response was detected under the N1 treatment, which may be explained by the lower application rate. Indeed, a meta-analysis of N-addition effects on CH_4_ fluxes showed that low application levels (less than 100 kg N ha⁻¹) tend to stimulate CH_4_ uptake, whereas higher doses result in inhibition [69]. As hypothesized, the fertilizer effect was very short term and had no influence on annual fluxes, as indicated by the lack of difference in emission rates among periods (table 4). An additional mechanism that may have influenced the observed soil CH_4_ fluxes is plant-mediated transport through oil palm stems. Previous work in oil palm plantations on peat has shown that CH_4_ can be transported from the peat through the palm and emitted via the stem, representing a potential pathway of ecosystem CH_4_ loss [36]. Because the present study quantified only soil surface fluxes, it is possible that part of the CH_4_ produced in the peat bypassed the soil surface and was released through the palms. Consequently, changes in palm physiological activity associated with fertilization or variations in water availability may have influenced the partitioning of CH_4_ between soil and stem emission pathways. For example, increased water and nutrient uptake following fertilization could potentially enhance plant-mediated CH_4_ transport, thereby reducing the amount of CH_4_ available for release through the soil surface. Measurements of stem CH_4_ fluxes would be required to evaluate this hypothesis.

Temporal variations in soil CH_4_ emissions were closely linked to fluctuations in the WT, although the observed relationship was opposite to expectations (table 5, eq. 8). Typically, CH_4_ emissions increase when the WT rises towards the surface, as oxygen availability declines and oxidation is reduced [14]. In contrast, our results revealed a negative relationship, which may be explained by an indirect effect of NO_3_^−^, given its strong positive correlation with WT level (supplementary material, §S6, supplementary material, figure S6, *p* < 0.0008). Interpretation of the WT effect should also be made with caution because WT level was measured only at discrete sampling times. Consequently, the measurements provide a snapshot of hydrological conditions but do not capture whether a given WT level was reached during a wetting or drying phase. Such hydrological hysteresis may influence microbial activity, CH_4_ production and CH_4_ oxidation, leading to different emission responses at the same measured WT level. Continuous WT monitoring would, therefore, be required to fully resolve the influence of hydrological dynamics on CH_4_ fluxes.

Finally, the relationships identified between CH_4_ fluxes and environmental variables provide insight into factors potentially influencing spatial and temporal variation in soil CH_4_ emissions. However, because observations were repeatedly collected through time, some temporal dependence among measurements may remain. Consequently, these relationships should be interpreted as indicative of potential environmental controls on CH_4_ dynamics, while recognizing that additional studies specifically designed to address repeated-measures structures would help further evaluate the relative importance of the identified drivers.

## 5. Conclusions

This study provides new insights into how nitrogen fertilization influences soil respiration and CH_4_ emissions in oil palm plantations established on tropical peat. Despite short-term responses to fertilizer addition, nitrogen application had no significant effect on annual soil respiration or CH_4_ fluxes under current management regimes. Annual soil respiration was remarkably high with pronounced spatial variability. The greater respiration observed FPs, corresponding with higher soil temperatures and N_2_O emissions, suggests spatial heterogeneity in peat mineralization rather than root-driven patterns. Annual CH_4_ emissions were relatively low across treatments but consistently higher in unfertilized plots, probably reflecting greater methanogenesis under reduced nitrate availability. The inverse relationship between CH_4_ fluxes and soil NO_3_^−^ concentrations supports the role of nitrogen cycling in regulating CH_4_ oxidation in these drained peat soils.

Overall, our findings highlight strong interactions between soil respiration, CH₄ fluxes and nitrogen dynamics in managed tropical peatlands. Spatial variability in fluxes and the coupling between C and N processes emphasize the need to account for within-plantation heterogeneity when assessing GHG emissions and refining process-based models. Improved mechanistic understanding of these interactions is essential for developing sustainable nutrient management strategies and accurately predicting the climate impacts of oil palm cultivation on peat.

## Supplementary Material



## Data Availability

The data that support the findings of this study are available at https://doi.org/10.17528/CIFOR/DATA.00314. Supplementary material is available online [70].
