# Supplementary material for: How does nitrogen application impact soil respiration and CH4 emissions? An experiment in an oil palm plantation on tropical peat
Source: R Soc Open Sci. 2026 Sep 9;13(9):rsos252443. doi: 10.1098/rsos.252443 (PMC13561043; doi:10.1098/rsos.252443)
Supplement: Supplementary file 1 [file rsos.252443.zip › rsos.252443/rsos252443_si_001.docx]

Supplementary material

**How does nitrogen application impact soil respiration and CH4 emissions? An experiment in an oil palm plantation fertilization trial on tropical peat**

Kristell Hergoualc’h^1, 2^^, 3^, Louis Verchot^4^, Satria Oktarita^1, 5^, Nelda Dezzeo^1, 6^

^1^ Center for International Forestry Research (CIFOR), Jl. CIFOR, Situ Gede, Bogor 16115, Indonesia

^2^ Centre de coopération International en Recherche Agronomique pour le Développement (CIRAD), UMR Eco&Sols, Montpellier, France

^3^ Mazingira Centre for Environmental Research and Education, International Livestock Research Institute (ILRI), Nairobi, Kenya

^4^ Center for International Tropical Agriculture (CIAT), Km 17, Recta Cali-Palmira, Cali, Colombia

^5^ Yayasan Hima Alam Raya, Kabupaten Lima Puluh Kota, West Sumatra, Indonesia

^6^ Venezuelan Institute of Scientific Research (IVIC), Caracas, Venezuela

**S1. Fertilization trial**

The trial comprised 54 plots each consisting of 32 palms (8 rows of 4 palms). The location of the studied plot is presented in Fig. S1.

**Fig. S1.** Location of the N0, N1 and N2 plots within the oil palm fertilization trial. Thick grey and blue lines surrounding the trial indicate the collection trails and secondary drainage canals, respectively. Thin lines within the trial are the tertiary drainage canals. The palms are represented by black dots.

**S2. Methods on CH_4_ flux rate determination**

When the concentration curve deviated from linearity, the flux was calculated using fewer sampling points (Fig. S2A). This occurs because the chamber introduces an artefact by reducing the concentration gradient between the soil and the atmosphere. Additionally, if any vial other than the one taken at chamber closure showed a concentration equal to ambient levels while the remaining samples exhibited a clear temporal trend, we assumed that the ambient-level vial had leaked (Fig. S2B). In such cases, the observation was excluded unless there was clear evidence at the time of analysis that the vial had remained properly pressurised. When no clear trend was observed, typically under conditions of zero or very low flux, the measurements were retained in the analysis.

**(B)**

**(A)**

**Fig. S2**. Case where the final observation of gas concentration was discarded from the analysis of the CH_4_ flux rate (A). Case where an observation equal to ambient CH_4_ concentration was discarded from the analysis of the CH_4_ flux rate (B).

**S3. Soil mineral N content, net mineralization and nitrification rates**

Nitrogen availability was measured in the absence of fertilization and one day following the application of 103 and 206 kg N ha^-1^ y^-1^ in the N1 and N2 treatments, at the CP position. The N0 treatment received no fertilizer.

**Table S3** Soil ammonium (NH_4_^+^) and nitrate (NO_3_^-^) contents, net mineralization and nitrification rates of the N0, N1 and N2 treatments in the oil palm plantation trial on peat. The results differentiate two spatial positions: close to palm (CP) and further from palm (FP) and two periods: without (February 2013, No Fert.) and after fertilizer application (April 2013, Fert.).

|  |  | N0 | N1 | N2 |
| --- | --- | --- | --- | --- |
|  |  | CP | | |
| No fert. | NH_4_^+^ (mg N kg^-1^) | 81.1 ± 28.9^ab^ | 108.2 ± 18.6^b^ | 24.8 ± 7.2^a^ |
|  | NO_3_^-^ (mg N kg^-1^) | 4.6 ± 3.5 | 3.5 ± 2.9 | 2.5 ± 0.6 |
|  | Net mineralization (mg N kg^-1^ d^-1^) | 3.5 ± 1.0 | 6.6 ± 2.8 | 3.8 ± 0.7^β^ |
|  | Net nitrification (mg N kg^-1^ d^-1^) | 1.1 ± 0.5 | 0.4 ± 0.2 | 0.1 ± 0.2 |
| Fert. | NH_4_^+^ (mg N kg^-1^) | 45.9 ± 15.1^a^ | 519.4 ± 361.8^ab^ | 1289.0 ± 629.9^b β^ |
|  | NO_3_^-^ (mg N kg^-1^) | 25.1 ± 4.5 | 28.7 ± 3.2 | 38.8 ± 7.8 |
|  | Net mineralization (mg N kg^-1^ d^-1^) | 2.6 ± 1.8 | 25.0 ± 32.8 | 36.4 ± 126.9 |
|  | Net nitrification (mg N kg^-1^ d^-1^) | 0.8 ± 0.6 | 0.9 ± 1.5 | 3.7 ± 3.1 |
|  |  | FP | | |
| No fert | NH_4_^+^ (mg N kg^-1^) | 97.7 ± 30.4^b^ | 121.1 ± 44.5^b^ | 10.7 ± 1.9^a^ |
|  | NO_3_^-^ (mg N kg^-1^) | 7.4 ± 2.8 | 7.7 ± 2.0 | 8.3 ± 3.1 |
|  | Net mineralization (mg N kg^-1^ d^-1^) | 6.0 ± 3.5 | 5.2 ± 1.7 | 1.4 ± 0.7^α^ |
|  | Net nitrification (mg N kg^-1^ d^-1^) | 0.7 ± 0.2^ab^ | 1.3 ± 0.4^b^ | 0.3 ± 0.2^a^ |
| Fert. | NH_4_^+^ (mg N kg^-1^) | 116.0 ± 53.5 | 93.3 ± 16.6 | 95.0 ± 17.5 ^α^ |
|  | NO_3_^-^ (mg N kg^-1^) | 25.8 ± 2.6 | 29.1 ± 3.4 | 33.1 ± 2.6 |
|  | Net mineralization (mg N kg^-1^ d^-1^) | -6.7 ± 6.1^a^ | -3.9 ± 0.8^a^ | 15.1 ± 12.9^b^ |
|  | Net nitrification (mg N kg^-1^ d^-1^) | -0.2 ± 0.6 | 0.1 ± 0.6 | 2.7 ± 2.0 |

Letters (a, b, c) indicate a significant difference between N treatments, within a spatial position. Symbols (α, β) indicate a significant difference between spatial positions, within an N treatment. In the absence of a significant difference no letters are displayed. Soil averages of mineral N content, mineralization and nitrification rates are based on five samples.

**S4. Magnitude of annual soil respiration and soil CH_4_ emissions in oil palm plantations on peat**

This review considered only studies with experimental designs capable of adequately capturing the spatiotemporal variability of soil respiration and CH_4_ emissions over a year. Eligible studies included at least three spatial replicates per site, monitored at a minimum frequency of every two months over a minimum of nine months. We compiled only soil emissions, excluding ditches and stem emissions, which were not monitored in our study. Data from Azizan *et al.* (2021) reporting unusually low soil respiration rates (2 Mg C ha⁻¹ y⁻¹) were excluded, as fluxes calculated from measurements at 0, 10, and 20 minutes after closure of large static chambers are known to underestimate peatland soil respiration.

**Fig. S4.** Boxplot summary of annual soil respiration (A) and CH_4_ (B) fluxes in oil palm plantations on peat. Circles with error bars indicate the annual cumulative fluxes from this study and their standard error (blue: N0, gray: N1, orange: N2). The review included 25 cases for soil respiration (b, c, d, e, f, g, h, i, j, l, m, n, o), 9 for CH_4_ (a, g, h, k, p). Study sources are: a, Azizan *et al.* (2021), Malaysia; b, Basuki *et al.* (2021), Indonesia; c, Chaddy *et al.* (2021), Malaysia; d, Comeau *et al.* (2013), Indonesia; e, Dariah *et al.* (2014), Indonesia; f, Ishikura *et al.* (2018), Malaysia; g, Lau *et al.* (2023), Malaysia; h, Manning *et al.* (2019), Malaysia; i, Marwanto and Agus (2014), Indonesia; j, Melling *et al.* (2005b), Malaysia; k, Melling *et al.* (2005a), Malaysia; l, Melling *et al.* ( 2013), Malaysia; m, Pulunggono *et al.* ( 2022), Indonesia; n, Sakata *et al.* (2015), Malaysia; o, Swails *et al.* (2019), Indonesia; p, Swails *et al.* (2021), Indonesia.

**S5. Relationship between annual soil respiration and soil C:N in oil palm plantations on peat**

**Fig. S5.** Relationship between annual soil respiration and soil C:N ratio in oil palm plantations on peat (blue: N0, gray: N1, orange: N2 treatments from this study). In addition to results from this research, the review included 20 for soil respiration cases from Chaddy *et al.* (2021), Comeau *et al.* (2013), Dariah *et al.* (2014), Ishikura *et al.* (2018), Lau *et al.* (2023), Melling *et al.* (2005b), Melling *et al.* (2013), Sakata *et al.* (2015), Swails *et al.* (2021).

**S6. Relationship between soil nitrate content and water table level**

**Fig. S6.** Relationship between soil nitrate content and water table level in the oil palm plantation fertilizer trial.

**References**

Azizan, S.N.F., Goto, Y., Doi, T., Kamardan, M.I.F., Hara, H., McTaggart, I., Kai, T., Noborio, K., 2021. Comparing GHG Emissions from Drained Oil Palm and Recovering Tropical Peatland Forests in Malaysia. Water 13, 3372.

Basuki, I., Kauffman, J.B., Peterson, J.T., Anshari, G.Z., Murdiyarso, D., 2021. Land Cover and Land Use Change Decreases Net Ecosystem Production in Tropical Peatlands of West Kalimantan, Indonesia. Forests 12, 1587.

Chaddy, A., Melling, L., Ishikura, K., Goh, K.J., Toma, Y., Hatano, R., 2021. Effects of Long-Term Nitrogen Fertilization and Ground Water Level Changes on Soil CO2 Fluxes from Oil Palm Plantation on Tropical Peatland. Atmosphere 12, 1340.

Comeau, L.-P., Hergoualc'h, K., Smith, J.U., Verchot, L.V., 2013. Conversion of intact peat swamp forest to oil palm plantation: Effects on soil CO2 fluxes in Jambi, Sumatra. Working Paper 110. CIFOR, Bogor, Indonesia.

Dariah, A., Marwanto, S., Agus, F., 2014. Root- and peat-based CO2 emissions from oil palm plantations. Mitig Adapt Strateg Glob Change 19, 831–843.

Ishikura, K., Hirano, T., Okimoto, Y., Hirata, R., Kiew, F., Melling, L., Aeries, E.B., Lo, K.S., Musin, K.K., Waili, J.W., Wong, G.X., Ishii, Y., 2018. Soil carbon dioxide emissions due to oxidative peat decomposition in an oil palm plantation on tropical peat. Agriculture, Ecosystems & Environment 254, 202-212.

Lau, S.Y.L., Midot, F., Dom, S.P., Lo, M.L., Chin, M.-Y., Jee, M.S., Yap, M.L., Chaddy, A., Melling, L., 2023. Application of ammonium sulfate affects greenhouse gases and microbial diversity of an oil palm plantation on tropical peat. Archives of Agronomy and Soil Science 69, 648-661.

Manning, F.C., Kho, L.K., Hill, T.C., Cornulier, T., Teh, Y.A., 2019. Carbon Emissions From Oil Palm Plantations on Peat Soil. Frontiers in Forests and Global Change 2.

Marwanto, S., Agus, F., 2014. Is CO2 flux from oil palm plantations on peatland controlled by soil moisture and/or soil and air temperatures? Mitigation and Adaptation Strategies for Global Change 19, 809-819.

Melling, L., Chaddy, A., Goh, K.J., Hatano, R., 2013. Soil CO2 fluxes from different ages of oil palm in tropical peatland of Sarawak, Malaysia as influenced by environmental and soil properties. Acta Hort. (ISHS) 982, 25-35.

Melling, L., Hatano, R., Goh, K.J., 2005a. Methane fluxes from three ecosystems in tropical peatland of Sarawak, Malaysia. Soil Biology & Biochemistry 37, 1445–1453.

Melling, L., Hatano, R., Goh, K.J., 2005b. Soil CO2 flux from three ecosystems in tropical peatland of Sarawak, Malaysia. Tellus 57B, 1–11.

Pulunggono, H.B., Siswanto, S., Mubarok, H., Widiastuti, H., Tambusai, N., Zulfajrin, M., Anwar, S., Taniwiryono, D., Sumawinata, B., Sabiham, S., 2022. Seasonal litter contribution to total peat respiration from drained tropical peat under mature oil palm plantation. Journal of Degraded and Mining Lands Management 9, 3247-3263.

Sakata, R., Shimada, S., Arai, H., Yoshioka, N., Yoshioka, R., Aoki, H., Inubushi, K., 2015. Effect of soil types and nitrogen fertilizer on nitrous oxide and carbon dioxide emissions in oil palm plantations. Soil Science and Plant Nutrition 61, 48-60.

Swails, E., Hergoualc’h, K., Verchot, L., Novita, N., Lawrence, D., 2021. Spatio-Temporal Variability of Peat CH4 and N2O Fluxes and Their Contribution to Peat GHG Budgets in Indonesian Forests and Oil Palm Plantations. Frontiers in Environmental Science 9, 1-15.

Swails, E., Yang, X., Asefi, S., Hergoualc’h, K., Verchot, L., McRoberts, R.E., Lawrence, D., 2019. Linking soil respiration and water table depth in tropical peatlands with remotely sensed changes in water storage from the gravity recovery and climate experiment. Mitigation and Adaptation Strategies for Global Change 24, 575-590.
